# A Balancing Act—20 Years of Nutrition and Health Claims Regulation in Europe: A Historical Perspective and Reflection

**DOI:** 10.3390/foods14091651

**Published:** 2025-05-07

**Authors:** Sonja Jost, Christian Herzig, Marc Birringer

**Affiliations:** 1 Center for Sustainable Food Systems, Justus Liebig University Giessen, Senckenbergstrasse 3, 35390 Giessen, Germany; christian.herzig@fb09.uni-giessen.de; 2 Department of Nutritional, Food and Consumer Sciences, Fulda University of Applied Sciences, 36037 Fulda, Germany; marc.birringer@oe.hs-fulda.de; 3 Relationship Management, University of Freiburg, Loewenstrasse 16, 79098 Freiburg, Germany

**Keywords:** claim-carrier, health claims, regulation, historical perspective, nutrient profiles, probiotics

## Abstract

The Nutrition and Health Claims Regulation (NHCR) has introduced a new regulatory perspective in food manufacturing, along with influencing consumers’ perception of health-related food claims. Since 2006, a new standard of science-based claims has significantly impacted the European health food market. Over the years, numerous additional decisions have been made, and the ongoing process remains challenging for policymakers striving to harmonize consumer protection and trade within and outside the European Union (EU). This paper presents the current state of the NHCR’s implementation, along with key events aimed at enhancing understanding among consumer organizations and food industry stakeholders, while also offering an insider perspective on relevant policy issues. Additionally, we address two pertinent policy issues to elucidate the associated challenges and opportunities, providing insights to support informed decision-making by policymakers. We use the nutrient profiles framework as a case study to illustrate considerations underpinning the objective of “consumer protection”, while the “probiotics” market serves as an example for exploring the goal of “facilitation of trade”. This historical perspective and reflection lead us to propose possible solutions for future food regulation.

## 1. Introduction

The understanding of food and its intricate relationship with health spans a rich historical tapestry. Across diverse cultures and time periods, various foods have been employed to promote and enhance humans’ health. Traditional practices, exemplified by the medicinal use of garlic in ancient Greek, Egyptian, and early American societies [1], underscore the enduring significance of natural ingredients. Concurrently, Traditional Chinese Medicine has garnered global recognition for incorporating plant-based ingredients into therapeutic formulations, steadily gaining popularity worldwide [2,3].

The commercialization of food, commencing in the 1900s, precipitated significant advancements in production and marketing. By the 1990s, a thriving market emerged, with probiotic yogurts, cereal bars, and protein shakes dominating, attracting consumers and reshaping dietary landscapes [4]. Health foods, or so called ‘functional foods’, began to gain more market value worldwide [5,6].

Although the industrialization of the food system supported the reduction in nutrient deficiencies [7], it simultaneously fostered the development and increasing prevalence of non-communicable diseases (NCDs) such as diabetes, cancer, or coronary heart disease, which currently constitute approximately “74 percent of all deaths globally” [8]. In 2019, poor diet ranked as the third highest risk factor contributing to the global burden of disease. In 2017, 22 percent of all adult deaths were associated with this factor [9]. To address these diseases, health foods appear to offer “quick-fix solutions” [10]. The often highly processed nature of these foods has made it both possible and necessary to regulate their sale and advertising to consumers. However, it is unsurprising that “the issue of healthy eating has a high political profile in many societies” [10].

Japan was the first country to refer to health foods as functional foods. With the regulation of Food for Specified Health Uses (FOSHU), it was the first country to establish a regulatory framework [11,12]. Between 1990 and 1997, the United States (US) established its framework [13], and the US Food and Drug Administration (FDA) was the first state organization to implement the term “health claims”. In 2025, the FDA updated its regulation on the nutrient content claim “healthy” on food packages [14].

In the late 1990s, the European Union (EU) started to establish projects such as FUFOSE (Functional Food Science in Europe) or PASSCLAIM (Process for the assessment of scientific support for claims on foods). They formed the basis for the EU’s Nutrition and Health Claims Regulation (NHCR), which was adopted in 2006 [15] and provides one of the strictest regulatory systems for functional foods worldwide [16].

Although the term “health claims” is used both in Europe and the US, and the overall regulatory purpose is similar—to prevent false marketing and protect consumers—their regulatory frameworks differ in several key aspects. One of the major differences lies in the nature of the claims permitted. In the US, health claims often emphasize reducing the risk of disease, whereas in Europe, most authorized health claims focus on supporting the normal physiological functions of the body. Another significant distinction is the authorization process. In the EU, all health claims must undergo scientific evaluation and approval by the European Food Safety Authority (EFSA). In contrast, the US has multiple types of authorization processes, of which only the Authorized Health Claims that meet the Significant Scientific Agreement (SSA) standard are comparable to the EU’s system [17,18].

Nevertheless, to this day, “the implementation of the Regulation [NHCR] remains incomplete” [19]; ongoing discussions on ‘nutrient profiles’ make consumer protection not fully possible and certain issues regarding specific product categories such as ‘probiotics’ make a facilitation of trade not possible, something which continues to concern lawmakers, consumer organizations, and food industry actors at both EU and national levels. Nutrient profiling is defined as “the science of classifying or ranking foods according to their nutritional composition for reasons related to preventing disease and promoting health” [20]. Nutrient profile models, such as Nutri-Score or the Keyhole symbol, are instrumental tools for assessing the nutritional quality of foods by incorporating information on their nutritional characteristics, in addition to the mandatory nutrition labeling. They are increasingly utilized by government bodies globally to establish nutrition-related policies. These models universally consider nutrients regarded as limits, including sodium, saturated fatty acids, and total sugars. Nutrient profiles exist in various forms and are developed by different stakeholders, resulting in considerable variation [21,22,23,24].

In the context of nutrient profiles in the EU and ongoing discussions, this article integrates considerations regarding front-of-pack nutrition labeling (FOPNL). While acknowledging that additional criteria for FOPNL exist, such as those associated with school food standards or the restriction of marketing to children [23,24], our primary focus lies on labeling and restricting claims. Therefore, FOPNL is regarded as one relevant form of nutrient profiling in order to protect consumers from misleading claims.

The second area continuously discussed with respect to the NHCR legislation concerns probiotic claims. Due to the long history of fermented milk products and consideration of gut health in Europe, this food category was initially a key driver for the European health food sector [25]. In the 1980s and 1990s, there was a growing trend of incorporating probiotic bacteria, primarily lactobacilli, into yogurts and fermented milks due to their recognized health advantages [26]. The industry capacity, as well as the consumer demand, or at least awareness, is high. It is estimated that the global probiotics market will grow from USD 57.8 billion in 2022 to USD 73.1 billion in 2025, reaching up to USD 85.4 billion by 2027 [27]. Although the probiotics market is expected to become more diverse, probiotic yogurts and fermented milk products will remain the largest sector [28]. Nevertheless, almost 20 years of regulation through the NHCR have influenced the sector and meanwhile many yogurts advertise health claims using vitamins or melatonin rather than relying on the potential beneficial effects of yogurt cultures [29,30]. In this overview, we will introduce the term “Claim-Carriers”, which explains this marketing-driven phenomenon. The legislation of probiotics is still unclear, not harmonized, and is starting to diverge in EU countries.

The aim of this article is to clarify the status quo of ‘health claims’ in Europe, considering the regulatory basis after the introduction of the NHCR in 2006 in the EU. An up-to-date literature search serves as the basis for the presented research. As examples, we present two cases, ‘nutrient profiles’ and ‘probiotic claims’, where the process of implementing the NHCR did not work as policymakers initially planned it. Those two issues serve as the basis for the discussion and conclusion.

## 2. Historical Outline on the Development of Nutrition and Health Claims Since 2006

The rules on nutrition and health claims were established in **2006** (Box 1). After the implementation of the NHCR into European law in **2007**, the workload of sorting applications and creating authorization procedures for all claims was substantial. Within the first six years, nutrition claims, which focus on the beneficial nutritional properties of foods, were sorted and defined with a list of 30 claims [31,32]. Since then, the list has remained fixed, and therefore, we will not consider it in the upcoming section.

Box 1 **Definitions according to NHCR Art.2, § 2.**

  “‘**Claim**’ means any message or representation, which is not mandatory under Community or national legislation, including pictorial, graphic or symbolic representation, in any form, which states, suggests or implies that a food has particular characteristics;”

  “‘**Nutrition claim**’ means any claim which states, suggests or implies that a food has particular beneficial nutritional properties (…).”

  “‘**Health claim**’ means any claim that states, suggests or implies that a relationship exists between a food category, a food or one of its constituents and health;”

**Figure 1:**
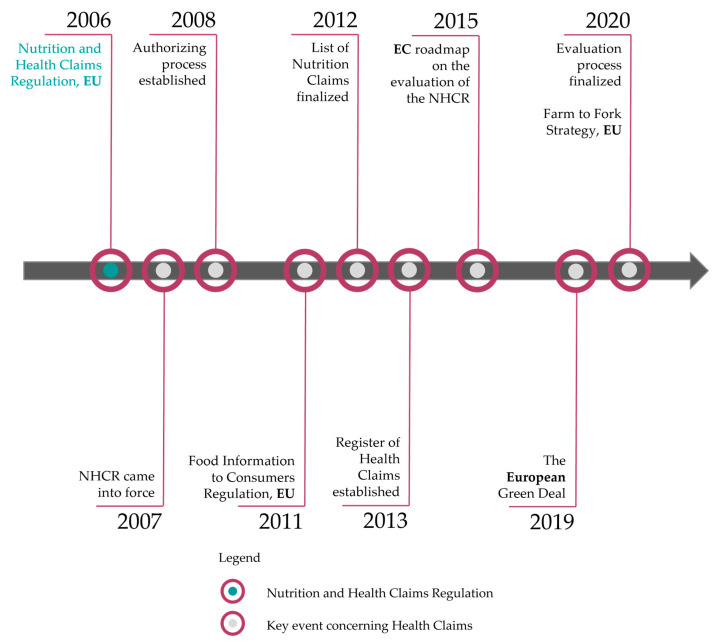
Timeline of key events with regard to the Nutrition and Health Claims Regulation including legend.

Several events have shaped the regulatory development of the NHCR; key milestones are outlined in the text and listed in Figure 1.

In **2008**, Commission Regulation (EU) No 353/2008 [33] established rules for applications regarding the authorization process of heath claims. In **2011**, a European Regulation focusing on Food Information to Consumers (FICR) came into force. Some elements overlapped because both regulations aim to properly educate and inform consumers by providing adequate and scientifically proven information on food products. Especially due to the current perspective on nutrient profiles, the FICR becomes an imminent factor in the discussion.

In **2012**, a list with 222 permitted health claims was established [34], leading to the EU Register of Health Claims online one year later [35]. In **2015**, the Commission published a Roadmap for evaluating the Nutrition and Health Claims legislation. The evaluation process was finalized in 2020 and continues to influence further discussions [36]. In 2019, the European Commission (EC) introduced ‘**The European Green Deal**’ with one of its goals being “improving people’s health” [37]. A concrete ‘**Farm to Fork Strategy**’ was proposed to guarantee its achievement [38].

### 2.1. The Setting of Nutrient Profiles

The NHCR stipulated the development of nutrient profiles (Article 4) by the European Commission until 2009. Nutrient profiles should enable consumers to make a healthy product choice by considering and understanding the nutritional value that a food possesses in its entirety. With that, the NHCR would tackle the threat of consumers buying unhealthy products bearing health claims, for example, with a high sugar content. **Nutrient profiles were envisioned to enable consumers to make a healthy decision with regard to a balanced diet**.

The European Parliament (EP) and the Council of the EU describe nutrient profiles as follows: “They [nutrient profiles] should be based on generally accepted scientific data relative to the relationship between diet and health. However, profiles should also allow for product innovation and should take into account the variability of dietary habits and traditions, and the fact that individual products may have an important role in the context of an overall diet” [15]. Article 4 of the NHCR even lists nutrient profiles as basis for “conditions for the use of nutrition and health claims” and defines them as follows. “These nutrient profiles (…) shall be established taking into account in particular:

- the quantities of certain nutrients and other substances contained in the food, such as fat, saturated fatty acids, trans-fatty acids, sugars and salt/sodium;
- the role and importance of the food (or of categories of foods) in the diet of the population in general or, as appropriate, of certain risk groups including children;
- the overall nutritional composition of the food and the presence of nutrients that have been scientifically recognized as having an effect on health.”

**Figure 2:**
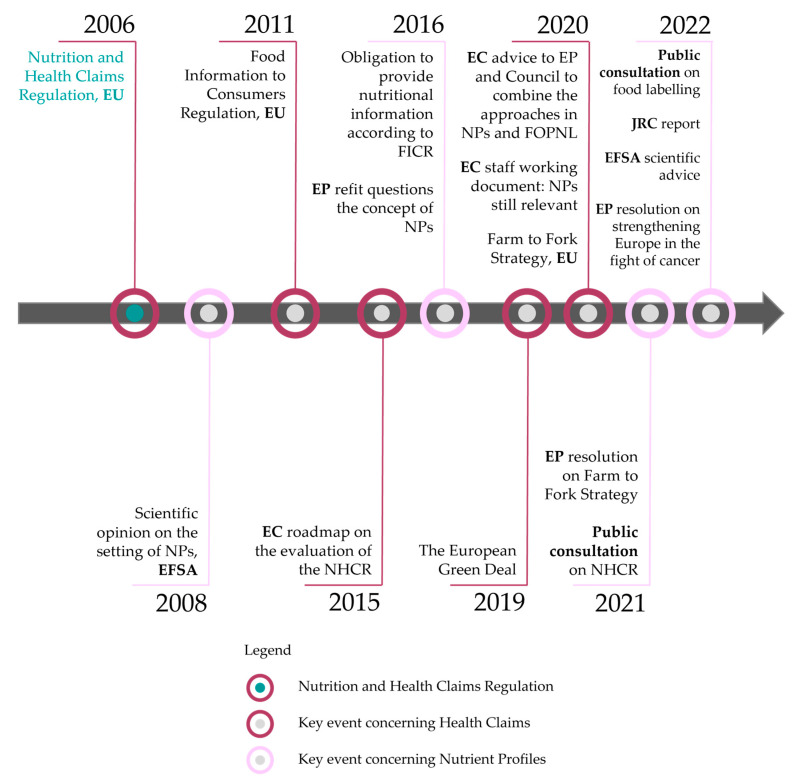
Timeline of key events with regard to nutrient profiles including legend.

Although they should have been established by **2009**, to date, nutrient profiles have not been implemented. Nevertheless, several steps have been taken in the past decade that should be considered and could contribute to resolving the issue of nutrient profiles, which can be found as key events in Figure 2.

In **2015**, the **EC** published a Roadmap on the Evaluation on the NHCR, which also focused on nutrient profiles. One of its key findings clarified that “(…) the setting of nutrient profiles is still pertinent and necessary (…)” [36,39].

One year later, the **EP** [40] published a statement questioning whether the concept of nutrient profiles could be eliminated due to “serious and persistent problems”, for example the “distortion of competition”. The common thread in both statements was the hope that the objective of establishing nutrient profiles, namely to enable consumers to make healthier food choices, could be achieved through the implementation of the FICR [41].

One aspect of the European Green Deal is the Farm to Fork Strategy [37]. This strategy aims to empower consumers to choose healthy diets, sparking a renewed discussion on the NHCR’s nutrient profiles, this time in conjunction with the nutritional information provided by the FICR [42].

In **2020**, the **EC** already advised the EP and the Council to consider “the strong link between nutrient profiling and FOPNL” and stated that nutrient profiles are still relevant [36,43]. The communication of the Farm to Fork Strategy commenced, emphasizing the necessity of nutrient profiles in addressing the healthiness of European consumers [38]. Subsequently, the **EP** stated that “nutrient profiles, which are long overdue, remain pertinent and necessary” [42].

In **2021** and **2022**, two **public consultation** processes were launched. The first, aimed at informing decisions on nutrient profiles, received 472 valid responses [44]. The second sought feedback on a new harmonized food labeling system designed to educate consumers, gathering 3225 valid responses. Additionally, the EC conducted targeted surveys among stakeholders, including consumer and business interest groups, companies of all sizes, and national authorities, yielding 200 responses. These inputs are expected to support the evaluation of a potential revision of the FICR [45].

With the focus on nutrient profiles, several significant developments occurred in **2022**:

- The **Joint Research Centre** released a report on food labeling, encompassing nutrient profiles and FOPNL [46].
- **European Food Safety Authority (EFSA)** issued updated scientific advice on nutrient profiles and FOPNL [47].
- The **European Parliament (EP)** published a resolution on bolstering Europe’s efforts against cancer, emphasizing the crucial need to “encourage and help consumers make informed, healthy and sustainable choices about food products” [48].

In **2025**, the EP published a document summarizing the discussions on FOPNL, concluding that discussions have to go on to ultimately find a solution that enables consumers to make healthier food choices. However, the topic remains divisive among member states, making a decision difficult [49].

### 2.2. The Legal Fit for Probiotics

In 2007, the Commission stated that “A claim is a health claim if in the naming of the substance or category of substances, there is a description or indication of a functionality or an implied effect on health”, and provided a direct example addressing probiotics: “contains probiotics/prebiotics—the reference to probiotic/prebiotic implies a health benefit.” Furthermore, they stated that “In the case of the claim ‘contains’, this means that the substance subject to the claim is present in a significant quantity and has been shown to have a beneficial nutritional or physiological effect” [50]. However, to date, EU policymakers have not defined probiotics further in relation to the NHCR. In its current state, the regulation does not even permit the provision of information to consumers about “probiotics as a category of ingredients” [51].

The definition provided by the Food and Agricultural Organization of the United Nations is most commonly used, defining probiotics as “Live microorganisms which when consumed in adequate amounts as part of food confer a health benefit on the host” [52].

To this day, the EFSA has assessed **355 probiotic claims** (Table 1), of which only **one claim has been scientifically accepted** within the broad spectrum of probiotics. One such claim asserts that “live cultures in yoghurt or fermented milk improve lactose digestion of the product in individuals who have difficulty digesting lactose” All other claims have not been authorized for marketing to consumers [35]. Table 1 summarizes by category all probiotic claims that were submitted and examined.

Following the question of whether it would be possible in the future to market probiotics as a **nutritional claim**, especially in food supplements, in **2017** [53], the **EC** reiterated its initial decision stating that “the term ‘probiotic’ is considered as implying a health benefit and should therefore be classified as a **health claim** and not as a nutrition claim. The EC is not intending to change this classification” [54]. The question on the marketing of probiotics in food supplements was posed by Piernicola Pedicini, an Italian Member of the European Parliament for the Group of the Greens/European Free Alliance. He further clarified that “In many Member States, the term ‘probiotic’ is widely understood as indicating a category of products. This is particularly true of Italy, one of the largest markets in Europe” [53].

His statement still underscores the currently fragmented situation regarding the regulation of probiotics under the NHCR. Italy, Spain, and France have legalized the use of the term ‘probiotic’ under specific conditions, allowing companies to advertise their products accordingly (Table 2).

**Figure 3:**
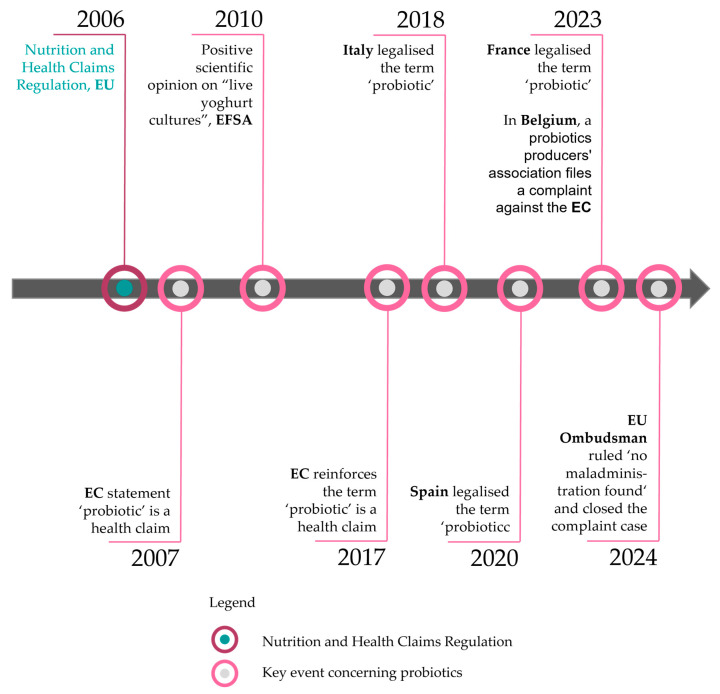
Timeline of key events with regard to probiotics including legend.

Italy, Spain, and France have a strong interest in addressing the matter of probiotics in the European Union. In 2016, Italy had the largest number of dairy companies in the EU, with 3830, followed by France with 1503, and Spain with 1485 [58]. Germany was the biggest market for milk substitutes in the EU in 2021, generating EUR 532 million in revenue, with Spain (EUR 445 million), Italy (EUR 291 million), and France (EUR 240 million) [59,60]. France led the dairy industry turnover in 2019 in the European Union, with approximately EUR 42 billion, with Germany (EUR 30 billion) and Italy (EUR 21 billion) following, and Spain in fifth place (EUR 9 billion) [61]. According to the International Probiotics Association Europe [62], Germany, France, Italy, and Spain are the leading probiotic markets in Europe. It is therefore not surprising that France, Spain, and Italy have introduced their own regulations on probiotics, which can be found as key events in Figure 3.

Other countries continuously mentioned by interest groups as well as press releases are Bulgaria, the Czech Republic, Denmark, Malta, the Netherlands, and Poland [63,64,65]. In order to be able to assess how the countries mentioned deal with probiotic claims, the authors contacted the respective authorities in the countries and asked for further information. The responses are listed in Table 3. Based on the responses, the authors of this article contradict the information that these countries have specific approaches to probiotics regarding health claims. Nevertheless, the use of the word ‘probiotic’ is often permitted on food supplements, either as an ingredient or categorical description.

In 2023, an antibiotics association filed a complaint in Belgium, arguing that the term ‘probiotics’ should be classified as a ‘nutrition claim’ rather than a ‘health claim.’ By the end of 2024, the European Ombudsman closed the case, concluding that the Commission’s interpretation of EU food legislation on probiotics is reasonable and aligns with its primary objective of ensuring high consumer protection [66].

## 3. Discussion

European regulators aimed to achieve two primary objectives with the implementation of the Nutrition and Health Claims Regulation (NHCR)—consumer protection and facilitation of trade (Box 2). However, upon examination of the implementation process, several issues emerge that deviate from the intended outcomes. Notably, claims related to caffeine attained positive health status, prompting concerns regarding consumer protection, particularly among vulnerable demographics such as adolescents. Consequently, the European Parliament ultimately rejected potential health claims to ensure safeguarding measures [67]. Similarly, claims regarding glucose were rejected by the European Commission to avoid encouraging consumers to consume foods high in sugar, as generally accepted nutritional and health principles advise against the consumption of high-sugar foods [68]. Additionally, botanical claims remain unregulated, presenting challenges related to the differentiation between dietary supplements (governed by food law) and the regulation of medicinal products or pharmaceuticals (governed by pharmaceutical law) [69].

Box 2 **Two main goals according to NHCR.**

  1. **Consumer protection:** “An increasing number of foods labelled and advertised in the Community bear nutrition and health claims. In order to ensure a high level of protection for consumers and to facilitate their choice, products put on the market, including imported products, should be safe and adequately labelled. **A varied and balanced diet is a prerequisite for good health and single products have a relative importance in the context of the total diet**” [70].

  2. Facilitation of trade: “Differences between national provisions relating to such claims may impede the free movement of foods and create unequal conditions of competition. They thus have a direct impact on the functioning of the internal market. It is therefore necessary to adopt Community rules on the use of nutrition and health claims on foods” [70].

In the discussion, the non-implementation of nutrient profiles as originally planned will serve as an example to illustrate how consumer protection could be enhanced. Furthermore, the ambiguous regulatory status of the probiotics market will be examined in terms of its opportunities and challenges for facilitating trade.

### 3.1. Nutrient Profiles—Challenges and Opportunities to Achieve Consumer Protection

The pressure to advance the matter of setting nutrient profiles has not only been advocated by policy advisors and organizations but also by numerous highly ranked scientific articles published within the last decade. For instance, Vos et al. [71] asserted that “A global shift towards more healthy and sustainable diets is necessary for the prevention of obesity and chronic diseases, as well as for the growing pressure on our ecosystems.”.

In 2022, the World Health Organization (WHO) published a review on the extent, nature, and effects of food marketing. The outcome is that unhealthy food is still being aggressively promoted, fostering the development of severe NCDs [72]. A healthy diet is at least one modifiable risk factor in addressing the burden of NCDs. Front-of-pack nutrition labeling (FOPNL) was identified as a possible key driver to enable consumers to make informed health choices [72]. Many models are already established or endorsed by governmental bodies [24,73,74]. Most countries in the Americas have already introduced mandatory FOPNL [75]. Nevertheless, it is not too late to take a leadership role by setting appropriate nutrient profiles across the European board to ensure the goal of protecting consumers from misleading claims.

To take on this leadership role, the European Union must address several challenges that still exist and need to be resolved to properly tackle the issue.

#### 3.1.1. Nutrient Profiles: Challenges

(a) EU institutions and authorities address different opinions

Looking back, in 2011, the Food Information to Consumers (FICR) came into law and sparked new discussions on nutrient profiles. In particular, the European Parliament raised concerns about whether nutrient profiles could live up to their expectations, with particular emphasis on the burden it placed on the industry [40].

In contrast, the European Commission was optimistic that the nutrition declaration that came with the FICR in 2016 would improve the situation for consumers. Additionally, they recognized that the establishment of nutrient profiles would not be easily settled but continued to emphasize its importance [39].

Several policy statements, such as the Farm to Fork Strategy, have been advancing the issue of nutrient profiles. Furthermore, several reports and public consultation processes have been conducted, with some of the results not yet published. This overwhelming flood of information exemplifies the complexity of the matter. With all the ongoing discussions and recommendations, it should be the goal of European policymakers to make a start and not be paralyzed by the various interests that have reached the EC through its various feedback rounds.

(b) Misleading marketing information on food products

With the current regulatory framework, food products with health claims are not necessarily healthy food choices. More than one-third of food products bearing claims, from categories such as breakfast cereals, juice drinks, and breads, are high in fat, sugar, and salt. To this day, it is possible for food companies to add ingredients with health claims just to use the claim for marketing purposes. We define such ingredients as “Claim-Carriers” (Claim-Carriers: Ingredients in foods with health claims that are only incorporated to implement the health claims; this might include fortified foods. Claim-Carriers are used for marketing purposes but simultaneously influence new product developments especially in the “health food” sector).

By implementing nutrient profiles within the framework of the NHRC, consumers should be empowered to make informed decisions regarding a balanced diet. Instead, the misuse of Claim-Carriers often misleads consumers and could foster a lack of trust in food regulation.

(c) Consumers’ buying decisions are not exclusively driven by health

Additionally, even when health claims are accurately employed within the scope of regulatory objectives, they may not necessarily encourage consumers to make healthy food choices [76,77,78]. Findings indicate that consumers tend to overestimate the health benefits of foods bearing health claims by interpreting that the food, in general, is healthy, rather than solely considering the specific health relationship of the claim [79]. This supports previous findings that showed no change in consumer perception when the wording of the claim was altered or restricted [79,80].

Studies on food characteristics influencing consumers’ buying motivation have shown that a health claim and its perceived healthiness is only one factor considered when buying food [81]. This effect is even more decisive when time for buying is limited [79] and also depends on the consumers’ knowledge [82]. Therefore, nutrient profiles could facilitate and expedite decision-making towards purchasing healthy foods. In this process of nudging, considerations should be made regarding specific consumer groups, such as parents who influence the dietary behaviors of their children; influencers; groups of different socioeconomic statuses, given the growing health disparities [71,83]; or the several million of European consumers with non-communicable diseases [84].

(d) Various labels on the European market

In the interim, several European countries have adopted various voluntary FOPNL schemes to additionally provide information to consumers:

- In Finland, the “Heart Symbol system” is utilized [85];
- In France, Germany, Spain, and others, the “Nutri-Score” has been implemented [73,86];
- Italy employs the “NutrInform Battery” scheme [87];
- The “Keyhole” symbol is used in EU Nordic member states such as Sweden or Denmark [75].

Due to the prolonged decision-making process, different labels have gained significant traction within their respective countries. Consequently, strong interests must now be considered and discussed before arriving at a European-wide decision [88]. This assertion is supported by Batista et al. [89], who observed a global array of FOPNL with distinct purposes. The inherent heterogeneity makes it impossible to designate a superior labeling scheme This polarization is recognized by the European Commission and used to justify the ongoing decision-making process, explaining why the Nutri-Score model cannot be supported as the superior model [49].

Although there are still some issues to be resolved, there are ongoing developments that show opportunities to enhance consumer protection by introducing proper nutrient profiles. It is clear that positive results can only be achieved with a comprehensive approach that includes education, public awareness campaigns, and policy incentives.

#### 3.1.2. Nutrient Profiles: Opportunities

(a) Nutrient profiles support healthy buying decisions

In their systematic review and meta-analysis considering FOPNL schemes, Corker et al. [90] concluded that FOPNL promotes healthy food choices. Additionally, prior studies have indicated that symbolic elements and the incorporation of color codes can aid consumers in assessing the healthiness of products [91,92]. This aspect is often taken into account in discussions regarding the establishment of FOPNL.

Consumers should be empowered to make informed choices by receiving accurate information in a clear and easily understandable manner [83,93]. Furthermore, it should be of interest to policymakers not only to consider food labeling but also information found elsewhere (e.g., on social media or the web) to positively influence food consumption [83].

Clearly, the voluntary information currently provided is insufficient to influence consumers’ healthy food choices and, consequently, to address the issue of food-related NCDs in Europe. The existing information is too complex, surpassing the time available for quick consumer decisions. Labels should be simplified, aligning with recommendations from the WHO [94].

Numerous stakeholders, including the WHO [72], the European Public Health Association [95], or the leading European Consumer Organisation BEUC [96], consistently advocate the implementation of nutrient profiles as an effective solution to empower consumers to make healthy food choices. This approach aims to reduce the financial burden associated with food-related NCDs.

(b) A European front-of-pack nutrition labeling scheme strengthens consumer trust

Additionally, implementing a single FOPNL system across all EU countries would enhance visibility and consumer confidence [71,97]. Standardization could also facilitate research opportunities and promote healthier nutritional choices among diverse populations [22,98]. This unified approach could serve as a Unique Selling Point, akin to the widely recognized and accepted EU organic label [99], thereby supporting informed choices for consumers.

In general, consumers express interest in relying on labels for their health [22]. According to a stakeholder involvement process conducted by the EC [45], which involved 3224 participants, including 65% EU citizens and 17% business-related respondents, the highest level of agreement was achieved for two statements regarding harmonization: “Food businesses should be subject to the same rules on FOPNL across the whole EU” (87%) and “Consumers should have access to the same FOPNL across the whole EU” (85%).

Given the diverse preferences and needs of consumers, various consumer education instruments should complement these developments, as there is no one-size-fits-all approach that can guarantee reaching every consumer [22].

(c) Specific nutrients can be considered to foster public heath

Concerning the nutrients that should be considered by policymakers, the EFSA has already provided scientific advice for the EU population, which aligns with most NP models worldwide—focusing on reducing saturated fatty acids, added sugars, and sodium. The WHO is against creating a positive list of nutrients within nutrient profiles labeling as it wishes to prevent food companies from using them to create “health halos” [94]. However, there is a pending matter within the EU regarding the increase in dietary fiber intake, which could be endorsed by nutrient profiles [24,47,73]. This suggestion aligns with the feedback from 85 percent of respondents who concurred that “Nutrition information on the front-of-pack should be consistent with dietary guidelines” [45].

(d) Nutrition policy should focus on consumers’ health

A significant turning point in the political discourse was reached with the unveiling of the EU’s Farm to Fork Strategy in 2020, underpinned by the European Green Deal of 2019. Once again, the health of the European consumer took center stage in discussions surrounding nutrition policy. In response to this political priority, the EC, EFSA, and JRC concurrently released a myriad of reports, with the EC additionally initiating various consultation and feedback processes. The outcome can be simplified and summarized as follows.

The introduction of EU-wide regulation on nutrient profiles would alleviate the burdens on food companies and ensure consistent information provision for EU citizens [44]. This is particularly crucial to address industry concerns, as well as consumer grievances raised by key interest groups FoodDrinkEurope and the European Consumer Organisation BEUC [62,97]. Ideally, such regulation would create a conducive environment for companies offering healthier food choices to consumers [44]. Moreover, incentives such as tax reductions could be extended to the food industry to encourage positive alterations in food formulations [100].

Additionally, nutrient profiles and FOPNL should be aligned [101], as FOPNL serves as a tool to support “the prevention of diet-related, non-communicable diseases like cardiovascular diseases, diabetes or cancers” [100]. Consumers would be better informed with an FOPNL [100]. Despite the European Commission’s intention to establish a unified FOPNL by the end of 2022 [102], this initiative has regrettably been postponed once again.

The implementation of effective policies remains crucial in bringing about significant dietary changes. Ultimately, the European Parliament finds itself at a critical juncture in determining the path forward. Previous decisions, such as the prohibition of scientifically proven health claims related to caffeine in products with high sugar content, underscore their dedication to prioritizing public health interests [67].

### 3.2. Probiotics—Challenges and Opportunities to Achieve Facilitation of Trade

Returning to the topic of probiotics, the situation for both the industry and consumers remains unclear. However, the reasons behind this lack of clarity are multifaceted, giving rise to several challenges that warrant consideration.

#### 3.2.1. Probiotics: Challenges

(a) An unregulated start fosters errors in the beginning of regulating

A crucial aspect to consider is that our research did not reveal any official statement from the European Commission explaining why the term ‘probiotic’ is linked to health benefits. Nevertheless, upon examining the period when the decision was made, compelling arguments emerge supporting the decision and shedding light on why the EC opted not to revise its stance, despite significant industry pressure.

A historical argumentation can be the development of probiotic products within Europe. In Italy, for example, food supplements with probiotics and vitamins claiming health benefits were already sold about 40 years ago [56]. By the 1990s, the market for probiotic yogurts, particularly in bottled dosages marketed as yogurt drinks such as Yakult, Actimel (Danone), and Nestlé LC1, emerged as one of the pioneering forces behind conventional functional foods (as opposed to food supplements) [26]. These products aggressively promoted health-enhancing slogans that were well-known among the general population [25].

(b) Safety concerns in a rapidly evolving probiotics market

Nowadays, safety concerns are becoming increasingly pertinent, prompting discussions on whether health benefits should be attributed to probiotic products. Traditionally, the probiotic sector emerged from the realm of fermented products; however, the rapid advancements in biotechnology and bioinformatics now enable the development of new probiotics from novel sources [103,104].

Safety concerns encompass various risks associated with probiotic consumption. Probiotics pose potential risks when taken in conjunction with medication, as they encode enzymes that can interact with drugs. Additionally, some probiotics may contribute to antibiotic resistance. Addressing safety issues is crucial, particularly as probiotics are often targeted towards vulnerable consumer groups. The quality of probiotics is paramount, especially considering the online market’s limitations for controls and sanctions, underscoring the need to ensure consumer protection [105].

The EFSA has outlined several reasons why probiotics fail to receive favorable scientific evaluations regarding health claims (Table 1). Some of them are crucial to guarantee consumers’ safety, such as the adequate characterization of the active microorganism. Some of these reasons are pivotal to ensuring consumer safety, such as the adequate characterization of active microorganisms. Another common reason for the failure of scientific substantiation of health claims is the insufficient description of the health benefits of probiotics. Changes in the composition of the gut microbiota are not a beneficial effect per se [106,107].

With the continual influx of new probiotic strains and products into the market, regulators must constantly update their evaluation criteria to accommodate emerging scientific research and technological advancements. This necessitates a dynamic regulatory framework capable of adapting to the rapid evolution of the probiotics industry.

(c) Impacts of a diverse landscape of probiotic regulation on the industry

The regulation of marketed beneficial microbes and probiotics varies significantly among countries, and the basic classification of probiotics is not globally harmonized [103,106]. This discrepancy may have influenced the decisions of member states to enact national legislation to circumvent the NHCR (Table 2).

Instead of improving the situation for consumers and food manufacturers, the diverse regulatory approaches might have led to supply chain disruptions and reduced economies of scale. Food manufacturers now have to consider various regulations, which influence aspects such as the labeling of products. This situation may create market entry barriers for small and medium-sized enterprises [108], which account for 99.2 percent of the European food industry market and generate 104 billion euros in added value [109].

In 2024, a new European Commission (EC) was formed, and a new European Parliament was elected. A new wave of lobbying is underway. Certainly, countries with a long history of and a high market capacity for probiotic products will bring this matter to the agenda of the EC and EP. This presents an opportunity for the newly formed EC and EP to assess the current situation and engage in discussions with relevant stakeholders on both consumers’ and manufacturers’ sides.

#### 3.2.2. Probiotics: Opportunities

(a) Prioritize the advancement of consumer protection in political agendas

The newly formed European Parliament and Commission have the opportunity to strengthen consumer protection, especially in light of the Ombudsman’s decision that the Commission’s interpretation of EU food legislation on probiotics is reasonable [66]. One goal should be to prevent consumers from the misleading use of Claim-Carriers on food products. By streamlining the regulation throughout the EU, consumers are enabled to make informed decisions. In particular, consumers’ interests should be actively considered and incorporated in a clarifying process, though this group is often underrepresented compared to the high number of industry actors actively lobbying for their interests [62].

(b) Clarification promotes harmonization

Drawing from the theory of new institutional economics, it is evident that regulatory frameworks lacking clarity and effective enforcement mechanisms often result in regulatory fragmentation and erosion [110,111]. Inconsistencies in the interpretation and implementation of regulations can lead to a breakdown in compliance and undermine the intended regulatory objectives. This phenomenon is particularly pronounced in areas where definitions and standards are ambiguous or subject to varied interpretations. Consequently, the EU should strive to prevent more countries from introducing their own probiotic regulations. Additionally, politicians should address this topic seriously with member states and interest group representatives. An initial step entails clarifying and harmonizing the use of the term ‘probiotic’ as an ingredient for food supplements and foods (Table 2 and Table 3).

Furthermore, tackling the complexities of regulatory fragmentation demands intensified efforts towards regulatory convergence and harmonization within the EU. By fostering closer collaboration among member states and streamlining regulatory procedures, Europe can foster a more unified and competitive food market. This underscores the pivotal role of EU policymakers in orchestrating inclusive dialogs and facilitating resolutions to the probiotic regulation dilemma.

## 4. Conclusions and Outlook

With the realization of the EU’s Nutrition and Health Claims Regulation (NHCR) in 2006 and its Register in 2013, the expectations of political actors and interest groups were high. With the new package of regulations and a defined procedure to realize a claim, the various actors aimed to streamline different aspirations into a set of rules to foster consumer protection and facilitate trade. Generally, regulations are a product of an increasingly complex and specialized environment, which was the case with the EU’s health food sector in the 1990s. A regulation should bring certainty and minimize transaction costs.

On the other hand, uncertainty nurtures individuality in the interpretation of legislation [112]. It was only a matter of time until EU member states would commence the establishment of their own nutrient profiles or devise national approaches to address the impending issue of probiotics. A comparable scenario is observed in the on-hold status of “botanicals” under the NHCR [113]. The absence of a reliable perspective is a pending concern that warrants serious consideration by EU institutions, particularly the EC and EP.

The status of probiotics presents uncertainties for both industry stakeholders and consumers. The lack of clarity regarding the association of the term ‘probiotic’ with health benefits, as observed in our research, raises fundamental questions. Despite industry pressure, to this day, the EC has chosen not to revise its stance.

The historical evolution of probiotics in Europe, particularly in Italy, highlights a longstanding presence of health claims with these products. Safety concerns today include potential interactions with medications and the risk of contributing to antibiotic resistance, especially for vulnerable consumer groups [105]. The EFSA underscores reasons for the failure of probiotics to receive positive scientific evaluations, emphasizing the need for clear characterization and benefits [106].

Navigating the complex landscape of nutrition and health in the scientific realm is undoubtedly challenging. The inherent lack of black and white distinctions requires a nuanced approach, placing the importance of contextualizing foods, particularly in relation to nutrient profiles, at the forefront. While scientific understanding evolves, investing in consumer education emerges as a potentially more effective strategy than solely focusing on specific food interventions.

Nevertheless, there are opportunities that should be explored by policymakers to ensure that market developments proceed in a controlled direction, focusing on a high level of consumer protection and providing reliable settings for food manufacturers simultaneously.

Political nudging proves to be a cost-effective avenue for promoting healthy food choices [114]. The need for regulatory alignment with current nutrition science is evident, exemplified by the European Union to fulfill its promise of implementing nutrient profiles, aligning with contemporary dietary guidelines. A parallel call is made by the FDA’s initiative to redefine the implied nutrient content claim of “healthy” [115].

The heightened consumer interest in health, as reflected in the “greater-than-ever” focus on self-care and wellness [116], necessitates a further developments of the marketing of health claims. Concurrently, educational initiatives for consumers become imperative, with the potential for front-of-pack nutrition labeling (FOPNL) to create buying motivation towards healthier food choices [22].

The European single market, facing the challenge of competitive distortion due to multiple established FOPNL, requires a judicious decision-making process. While an ideal scenario would involve a singular FOPNL, the pragmatic choice may be to empower EU’s National Competent Authorities to select from existing labels. The underlying rationale is clear—the absence of any label impedes informed consumer choices, contributing to persistently high rates of NCDs and consequent financial burdens on the EU.

An analysis of existing health taxes on food based on nutrient profile models reveals only a few examples, such as Hungary’s Public Health Product Tax and Mexico’s tax on non-essential energy-dense foods. The WHO emphasizes that, unlike tobacco taxation, health taxes on food have the power to shape markets significantly through the strategic use of tiers and thresholds, sending strong signals to consumers as well as driving industry action. Consumer awareness and understanding of taxes (salience) are identified as critical factors [94]. The anticipated implementation of the Diet Impact Assessment Tool by the WHO might bring about substantial changes in this domain [117].

In the face of complexity, decision-making becomes an unavoidable imperative. The continuous balancing act between the inability to act due to complexity and the absolute necessity of making decisions underscores the intricate nature of addressing health and nutrition challenges. In this dynamic landscape, a comprehensive and adaptable strategy, encompassing both education and regulation, emerges as crucial for fostering a healthier future.

Both consumers and food manufacturers have the potential to shape the landscape of the Nutrition and Health Claims Regulation by actively participating in discussions through their representative interest groups. It is their responsibility to bring critical issues to the political agenda and assume a guiding role in the policy process.

## Figures and Tables

**Table 1:** List of categorized probiotic claims in the EU Register of Health Claims.

Probiotic Nutrient Substance	Number of Claims
*Lactobacillus*	157
*Bifidobacterium*	63
*Streptococcus*	12
Combination of two or more probiotic bacteria strains	86
Others	37
Total probiotic claims	355

**Table 2:** Overview of specific regulation for probiotic claims the EU.

Country	Probiotics in Food	Probiotics in Food Supplement	Year of Specific Probiotic Statement
France [55]	considered as a “non-specific health claim” which is allowed if accompanied by a specific authorized claim related to the probiotic action	Allowed as a category name to characterize the nature of the substances used in the product	2024
Italy [56]	Requirements: 1. traditional use 2. safe 3. being active in the intestines in a specific quantity to multiply	Same as for food	2018
Spain [57]	Term ‘probiotic’ can be labeled on foods but not as a health claim until decided a uniform approach within the EU	Same as for food	2020

**Table 3:** Overview of specific regulation for probiotic claims the EU—statements received on request.

Country	Specific National Practice on Probiotics	Specific Provisions for Food Supplements
Bulgaria	no response to the request	
Czech Republic	No	Not specified
Denmark	Yes, food supplements	Term ‘probiotic’ can be used as mandatory category designation for dietary supplements Only in the labeling Not allowed in ingredient list No health claim allowed
Malta	no response to the request	
Netherlands	Yes, food supplements	Term ‘probiotic’ can be used as an indicator of a category in food supplements
Poland	Yes, food supplements	Term ‘probiotic’ is accepted in labeling of food supplements as information that is required under provisions of Directive 2002/46/EC No health suggestions are allowed

## Data Availability

The original contributions presented in this study are included in the article. Further inquiries can be directed to the corresponding author.

## Abbreviations

The following abbreviations are used in this manuscript:

BEUC	The European Consumer Organization
EC	European Commission
EFSA	European Food Safety Authority
EP	European Parliament
EU	European Union
FDA	US Food and Drug Administration
FICR	Food Information to Consumers Regulation
FOPNL	front-of-pack nutrition labeling
FOSHU	Food for Specified Health Uses
FUFOSE	Functional Food Science in Europe
NCDs	non-communicable diseases
NHCR	EU’s Nutrition and Health Claims Regulation
PASSCLAIM	Process for the assessment of scientific support for claims on foods
U. S.	United States
WHO	World Health Organization
